# A new method for phase-pure χ-Fe_5_C_2_ synthesis to obtain linear α-olefins

**DOI:** 10.1093/nsr/nwaf018

**Published:** 2025-01-22

**Authors:** Jie Yan, Ding Ma

**Affiliations:** Beijing National Laboratory for Molecular Sciences, New Cornerstone Science Laboratory, College of Chemistry and Molecular Engineering, Peking University, China; Beijing National Laboratory for Molecular Sciences, New Cornerstone Science Laboratory, College of Chemistry and Molecular Engineering, Peking University, China

Linear α-olefins (LAOs) are essential chemical intermediates for the production of a wide range of high-value chemicals and materials, and are currently obtained by ethylene oligomerization. The Fischer–Tropsch (FT) process offers an alternative route to produce LAOs directly from syngas (a mixture of CO and H_2_) derived from carbon-based feedstocks such as natural gas, coal and biomass. However, the current syngas-to-LAOs process generates significant amounts of CO_2_ as a by-product, which is not only a concern from a greenhouse gas emission perspective, but also reduces the overall carbon efficiency of the process. Fe_5_C_2_ has been found to be highly active for conversion of syngas to LAOs [1,2]. Recently, Wang *et al*. developed a new method for the synthesis of a phase-pure χ-Fe_5_C_2_ catalyst, which significantly improves both the activity and selectivity of syngas to LAOs while minimizing CO_2_ production [3].

**Figure 1.**
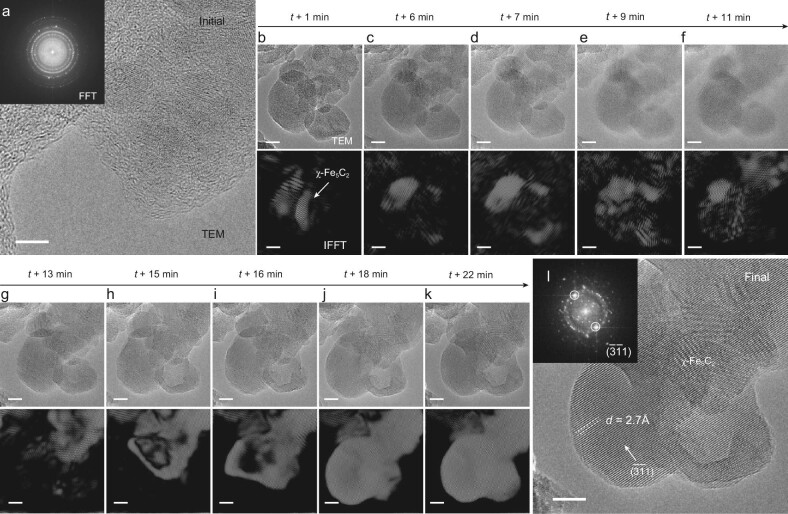
Environmental TEM study of phase-pure χ-carbide formation. (a–l) High-resolution TEM images of the initial state of Raney Fe (a), during carburization (b–k) and the final state (l). The corresponding inverse fast Fourier transform (IFFT) images show the location of χ-carbide in real space (environmental TEM conditions: H_2_/CO = 30, 1200 Pa, 350°C). Scale bars, 5 nm. Reprinted from Ref. [3].

The authors have provided a unique and concise method for the preparation of phase-pure χ-Fe-carbide, enabling the synthesis of 100% pure χ-Fe-carbide for the first time. The formation of phase-pure χ-carbide from passivated Raney Fe was observed by environmental transmission electron microscopy (TEM), as shown in Fig. 1. This synthesis method effectively avoids excessive carbon build-up on the catalyst surface, resulting in unusually high catalytic activity. In previous work, it was shown that phase-pure ε-carbide displays a low primary CO_2_ selectivity due to the absence of Fe-oxides [4]. Primary CO_2_ formation mainly depends on intrinsic properties of the catalyst, while secondary CO_2_ is formed *via* the water-gas-shift reaction and is mainly determined by the H_2_O partial pressure, which depends on CO conversion. Similar to phase-pure ε-carbide, phase-pure χ-carbide produced negligible primary CO_2_. Although secondary CO_2_ was not completely eliminated, the overall CO_2_ selectivity was drastically reduced, significantly improving carbon efficiency.

After being promoted with Mn, the phase-pure χ-Fe_5_C_2_ catalyst shows higher CO conversion and lower CO_2_ selectivity under industrially relevant conditions at 250°C than the catalysts reported in the literature at higher temperatures (>320°C) [5]. The low CO_2_ selectivity of the catalyst implies a high selectivity to desired LAOs, and the catalyst was able to produce the desired C_2_–C_10_ LAOs with 51% carbon-based selectivity while producing only 9% CO_2_. Further, at 290°C, the Mn/χ-Fe_5_C_2_ catalyst showed 1–2 orders of magnitude higher activity than that of conventional FT catalysts and remained stable for 200 hours, showing high industrial potential.

The development of the phase-pure χ-Fe_5_C_2_ catalyst represents a significant advancement in the field of C_1_ chemistry, particularly for the production of LAOs from syngas. The authors designed and synthesized new phase-pure χ-carbide catalytic materials, which provide an original high-carbon efficiency, low CO_2_ selectivity and high-activity technological route for the direct production of LAOs from syngas, and open the gate of high-carbon efficiency for the direct production of high-end chemicals from syngas. Future work may focus on exploring its applicability in industrial-scale processes. Researchers can also maximize the potential of this catalyst for other C_1_ chemistry applications, such as the production of alcohols, aromatics, or other types of olefins.
